# Cone Photoreceptor Degeneration and Neuroinflammation in the Zebrafish Bardet-Biedl Syndrome 2 (*bbs2*) Mutant Does Not Lead to Retinal Regeneration

**DOI:** 10.3389/fcell.2020.578528

**Published:** 2020-11-26

**Authors:** Ping Song, Joseph Fogerty, Lauren T. Cianciolo, Rachel Stupay, Brian D. Perkins

**Affiliations:** ^1^Department of Ophthalmic Research, Cole Eye Institute, Cleveland Clinic, Cleveland, OH, United States; ^2^Department of Ophthalmology, Cleveland Clinic Lerner College of Medicine, Case Western Reserve University, Cleveland, OH, United States; ^3^Department of Molecular Medicine, Cleveland Clinic Lerner College of Medicine, Case Western Reserve University, Cleveland, OH, United States

**Keywords:** cilia, BBSome, regeneration, zebrafish, Bbs2, Müller cell

## Abstract

Bardet-Biedl syndrome (BBS) is a heterogeneous and pleiotropic autosomal recessive disorder characterized by obesity, retinal degeneration, polydactyly, renal dysfunction, and mental retardation. BBS results from defects in primary and sensory cilia. Mutations in 21 genes have been linked to BBS and proteins encoded by 8 of these genes form a multiprotein complex termed the BBSome. Mutations in *BBS2*, a component of the BBSome, result in BBS as well as non-syndromic retinal degeneration in humans and rod degeneration in mice, but the role of BBS2 in cone photoreceptor survival is not clear. We used zebrafish *bbs2^–/–^* mutants to better understand how loss of *bbs2* leads to photoreceptor degeneration. Zebrafish *bbs2^–/–^* mutants exhibited impaired visual function as larvae and adult zebrafish underwent progressive cone photoreceptor degeneration. Cone degeneration was accompanied by increased numbers of activated microglia, indicating an inflammatory response. Zebrafish exhibit a robust ability to regenerate lost photoreceptors following retinal damage, yet cone degeneration and inflammation was insufficient to trigger robust Müller cell proliferation. In contrast, high intensity light damage stimulated Müller cell proliferation and photoreceptor regeneration in both wild-type and *bbs2^–/–^* mutants, although the *bbs2^–/–^* mutants could only restore cones to pre-damaged densities. In summary, these findings suggest that cone degeneration leads to an inflammatory response in the retina and that BBS2 is necessary for cone survival. The zebrafish *bbs2* mutant also represents an ideal model to identify mechanisms that will enhance retinal regeneration in degenerating diseases.

## Introduction

Bardet-Biedl Syndrome (BBS) is a pleiotropic, autosomal recessive disorder that is genetically and clinically heterogeneous (Beales et al., 1999; Weihbrecht et al., 2017). BBS is a ciliopathy and the primary features of BBS include obesity, retinal degeneration, cognitive impairment, postaxial polydactyly, renal abnormalities, and hypogonadism.

Mutations in more than 20 different genes cause BBS (Weihbrecht et al., 2017). A subset of the proteins encoded by *BBS* genes (Bbs1, Bbs2, Bbs4, Bbs5, Bbs7, Bbs8, Bbs9, and Bbs18) assemble into an octomeric protein complex called the BBSome (Nachury et al., 2007; Nachury, 2018). The BBSome facilitates cargo movement through cilia through multiple mechanisms. By interacting with the Intraflagellar Transport (IFT) complex, the BBSome serves as an adaptor to expand the pool of possible cargos (Liu and Lechtreck, 2018) and to facilitate exit of G-protein coupled receptors (GPCRs) from the ciliary membrane (Datta et al., 2015; Ye et al., 2018).

Retinal dystrophy is associated with greater than 95% of all patients and BBS proteins play a critical role in photoreceptor morphogenesis and survival (Hsu et al., 2017; Weihbrecht et al., 2017). Humans and mice lacking Bbs function exhibit retinal degeneration and photoreceptor loss (Fulton et al., 1993; Abd-El-Barr et al., 2007; Davis et al., 2007; Zhang et al., 2011, 2013; Dilan et al., 2018). In photoreceptors, protein trafficking is essential for the elaboration and growth of the outer segment (OS). The outer segment is a modified sensory cilium and utilizes the same mechanisms of protein trafficking as primary cilia (Insinna and Besharse, 2008). The outer segment of photoreceptors contains hundreds of tightly stacked disk membranes containing the proteins required for phototransduction. These disks are shed from the OS tips after approximately 10 days as new disks are formed at the base of the outer segment (Young, 1967). Proper disk formation and outer segment growth requires ciliary trafficking and BBSome activity (Hsu et al., 2017; Dilan et al., 2018). The primary GPCR in rod photoreceptors is rhodopsin and the cone opsins are GPCRs for the cone photoreceptors. Unlike in primary cilia, however, a role for the BBSome in GPCR trafficking in photoreceptors is questionable (Dilan et al., 2018). Rhodopsin and cone opsins do not migrate between the OS and the plasma membrane and there is little evidence that disruption of the BBSome results in a significant block in rhodopsin trafficking (Seo and Datta, 2017). While the majority of studies have focused on the effects of BBSome disruption to rod photoreceptors, less is known about the role of the BBSome in cones. Furthermore, evidence from mouse has suggested that photoreceptor function and morphology can be rescued if BBSome function is restored within an early window (Hsu et al., 2017). A prior characterization of *Bbs2*-null mice documented a slow retinal degeneration and other defects that resemble BBS in humans (Nishimura et al., 2004). While rhodopsin mislocalization was reported, the effects on cones was not investigated. It is important, therefore, to consider how photoreceptors, particularly cones, are impacted by loss of Bbs2 and how cones may be rescued or restored through regeneration.

We now report on a zebrafish model of *bbs2*. Zebrafish are cone-dominant, freshwater fish that have the ability to regenerate lost photoreceptors following acute injury (Goldman, 2014), but few studies have examined regeneration in the context of a progressive disease model in zebrafish. We report that zebrafish lacking *bbs2* exhibit early signs of visual dysfunction and undergo a slow, progressive degeneration of cones. Degeneration is accompanied by an inflammatory response from the microglia but not reactive gliosis by Müller cells. Importantly, acute injury stimulates Müller glia proliferation and modest regeneration of cones.

## Materials and Methods

### Animal Maintenance

Adult zebrafish were maintained and housed in 1.5, 3.0, and 10 L tanks in an Aquatic Habitats recirculating system (Pentair; Apopka, FL, United States) as previously described (Lessieur et al., 2019).

### Ethics Statement

All animal procedures were done with approval by the Institutional Animal Care and Use Committee (IACUC) at the Cleveland Clinic. Experiments were conducted according to relevant guidelines and regulations.

### CRISPR/Cas9 Gene Editing

Potential CRISPR target sites were identified in exon 4 of the zebrafish *bbs2* gene using ZiFiT^1^ (Sander et al., 2010) and gRNA synthesis was performed according to the protocol by Talbot and Amacher (Talbot and Amacher, 2014). Briefly, oligonucleotides for gRNA synthesis (5′ TAGGAGGAAACTGTGCTCTTCA 3′ and 5′ AAACTGAAGAGCACAGTTTCCT 3′) were annealed and ligated into plasmid pDR274 (Addgene, Watertown, MA, a gift from Keith Joung). Purified plasmid DNA was digested with *Dra*I (New England BioLabs, Beverly, MA) and used for *in vitro* transcription reaction to generate gRNA. Zebrafish embryos were injected at the 1-cell stage with a 1 nL solution of *bbs2* gRNA (200 ng/μL) and Cas9 protein (10 μM; New England BioLabs, Beverly, MA). Mutagenesis was confirmed by High Resolution Melt Analysis (HRMA) in injected F_0_ animals at 3 days post fertilization (dpf). Remaining F_0_ injected animals were raised to adulthood and outcrossed to wild-type fish and HRMA was performed on DNA from F_1_ progeny to screen for mutations. F_0_ founders producing a high degree of mutant F_1_ progeny were kept and individual *bbs2* alleles were identified by sequencing.

### Optokinetic response (OKR) Behavior Assay

OKR assays were performed on larval zebrafish as previously described (Lessieur et al., 2019). Briefly, 5–6 dpf larvae were immobilized in 35-mm petri dishes containing 4% methylcellulose and oriented using a dissecting needle. Dishes were placed inside the VisioTracker (VisioTracker 302060 Series, TSE Systems, GmbH Bad Homburg, Germany) and assayed for both contrast sensitivity and spatial frequency response functions (Rinner et al., 2005). All tests were conducted between 12 and 6 p.m. to avoid circadian variation (Emran et al., 2010). The genotype of each animal was verified by PCR following the OKR tests.

### Histology

Light-adapted larvae were prepared for histology as previously described (Lessieur et al., 2019). Briefly, larvae were anesthetized and bisected through the swim bladder. The heads were prepared for electron microscopy and the tails were used for genotyping. Fixation, plastic embedding, and electron microscopy were done as previously described (Lessieur et al., 2019).

### Immunohistochemistry

Zebrafish larvae were euthanized on ice and subsequently fixed in 4% paraformaldehyde, equilibrated in PBS + 30% sucrose for 2 h. After washing with PBS, larvae were embedded in Tissue Freezing Medium (Electron Microscopy Sciences, Hatfield, PA). Adult zebrafish were deeply anesthetized with tricaine methanesulfonate (0.4 mg/mL) and decapitated with a razor blade. Eyes were enucleated and fixed for 2 h in 4% paraformaldehyde. Eyes were washed in PBS and equilibrated in 5% sucrose/PBS for 3 h at room temperature and then in 30% sucrose overnight at 4°C. Eyes were washed in a 1:1 solution of 30% sucrose:Tissue Freezing Medium overnight at 4°C and subsequently embedded for cryosectioning.

Transverse cryosections sections (10 μm) were cut on Leica CM1950 cryostat and mounted on Superfrost Plus slides and dried at room temperature overnight. Slides were washed 3 × 10 min in PBS and then incubated in blocking solution (PBS + 2% BSA, 5% goat serum, 0.1% Tween-20, 0.1% DMSO) for 1 h. The following primary antibodies were used: mouse monoclonal zpr1 (1:100, Zebrafish International Resource Center (ZIRC), Eugene, OR, United States), mouse monoclonal zpr3 (1:100, ZIRC), mouse monoclonal 4C4 (1:1,000, a gift from Dr. Peter Hitchcock, University of Michigan), rabbit polyclonal L-plastin (1:1,000, GeneTex, Irvine, CA, United States, GTX124420), mouse monoclonal PCNA (1:100, Sigma, St. Louis, MO, United States, clone PC-10), rabbit polyclonal syntanxin3 (1:100; Synaptic Systems, Atlanta, GA, United States), Peanut agglutinin (PNA)-lectin conjugated to Alexa-568 (1:100, Thermo Fisher Scientific, Waltham, MA, United States). EdU labeling was detected with the Click-iTEdu Alexa Fluor-555 Imaging Kit (Thermo Fisher Scientific). Alexa-conjugated secondary antibodies were used at 1:500 in blocking buffer and incubated for at least 1 h. Slides were counterstained with 4, 5-diamidino-2-phenylendole (DAPI) to stain nuclei. Sections were imaged on a Zeiss Imager Z.2 equipped with an ApoTome using Zen2 software and post-processed in ImageJ. All analysis was performed only on sections that included or were immediately adjacent to the optic nerve.

### Light Damage

Light damage experiments were performed using a protocol adapted from Thomas and Thummel (Thomas and Thummel, 2013). Adult zebrafish were first dark adapted for 36–42 h. Up to 5 animals were placed in a 250 mL glass beaker with system water that was seated inside a 1L glass beaker with Milli-Q water and exposed to high-intensity light from a 120W X-CITE series 120Q metal halide lamp (Excelitas) for 30 min and then exposed to 14,000 lux light from a illumination cage for 4 h. Animals were allowed to recover in system water for 3 or 30 days. To label proliferating cells during early stages of regeneration, animals were injected intraperitoneally with 20 μL of a 20 mM EdU solution (PBS) at 2 days post injury and eyes were enucleated 24 h later (3 days post injury). To assess regeneration, animals were allowed to recover for 30 days post injury prior to enucleation. Retinas were processed for immunohistochemistry as described above.

### Statistics and Data Analysis

All data was analyzed and graphed using GraphPad Prism (v8). OKR data was analyzed by two-way ANOVA with Sidak corrections for multiple comparisons. Between 9 and 13 animals were tested by OKR. Cone outer segment density was quantified by counting PNA + outer segments and measuring the curvilinear distance of retina in ImageJ. Outer segment counts were analyzed in Prism8 by using one-way Brown-Forsythe and Welch ANOVA tests with Dunnett T3 corrections for multiple comparisons. Sample sizes are provided for each experiment.

## Results

### Generation of a Zebrafish *bbs2* Mutant

**FIGURE 1:**
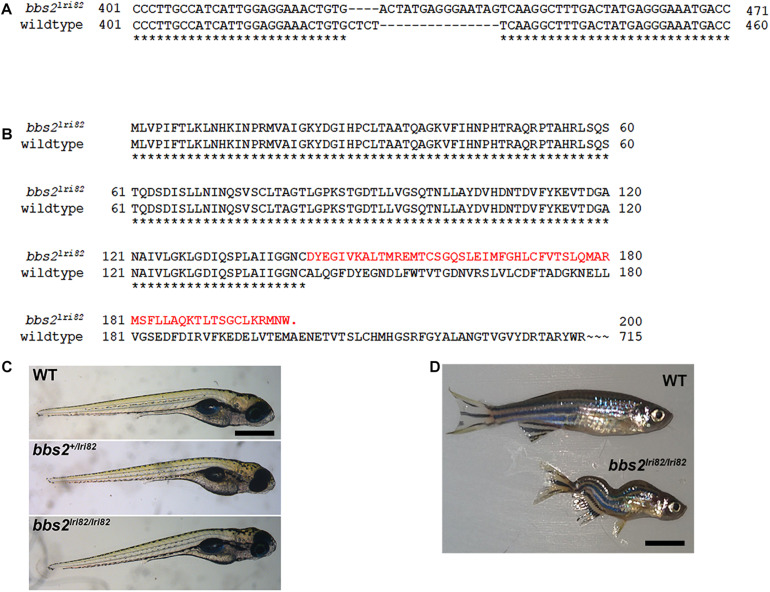
Mutation in zebrafish *bbs2* exon 4 causes a frame shift and premature truncation. **(A)** Coding sequence alignment of the wild-type and *bbs2^*lri*82^* alleles. A 15 bp fragment replaces “CTCT” from the wild-type allele, yielding a net 11-bp insertion and a frame shift. Numbering is relative to the start codon. **(B)** Amino acid sequences the of the wild-type and *bbs2^*lri*82^* peptides. The *bbs2^*lri*82^* mutation results in a frame shift that terminates after 58 codons (red). **(C)** Lateral view of 5 dpf wild-type (top), *bbs2^+/lri82^* heterozygotes (middle) and *bbs2^*lri*82/lri82^* homozygotes (bottom) that were verified by genotyping. No differences could be observed. **(D)** Lateral view of a 10 month old wild-type (top) and *bbs2^– /–^* homozygous mutant (bottom). Mutants are smaller and exhibit spinal curvature as adults. Scale bar: **(C)** 400 μm; **(D)** 7 mm.

To assess the role of *BBS2* in photoreceptor function, we utilized the CRISPR/Cas9 gene editing system to target exon 4 of the zebrafish *bbs2* gene. One mutant line (*lri82*) was recovered that resulted in the insertion of a 15-base pair fragment combined with a 4-bp deletion, thereby yielding an 11-bp insertion and a frameshift with a premature stop codon (Figure 1A). The truncated protein is predicted to contain the first 30% of the WD40 domain and then terminate after an additional frameshifted 58 amino acids (Figure 1B). Founder (F_0_) animals were outcrossed to wild-type animals to generate *bbs2* heterozygote (*bbs*2^ + /−^) zebrafish. Genotyping of clutches from *bbs*2^ + /−^ intercrosses revealed that homozygous offspring (*bbs2^–/–^*) appeared normal and were present in Mendelian ratios at 5 days post-fertilization (dpf) (Figure 1C). *bbs2^–/–^* mutants survived to at least 1 year of age, but developed spinal curvatures and were smaller than *bbs*2^ + /−^ and wild-type siblings (Figure 1D). These phenotypes resemble those of other zebrafish ciliopathy mutants (Lessieur et al., 2019).

### Zebrafish *bbs2* Mutants Have Impaired Visual Function at 5 dpf

**FIGURE 2:**
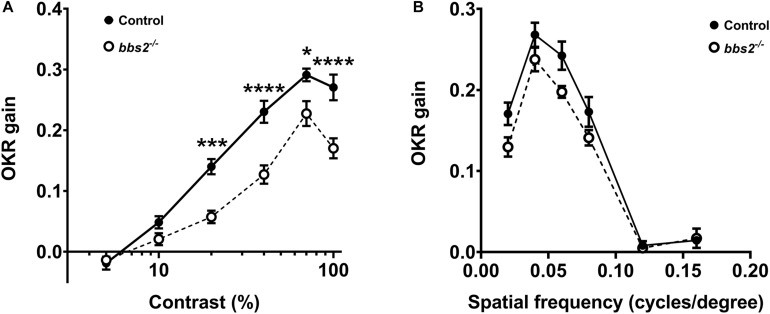
*bbs2 ^–/–^* mutant zebrafish have visual function deficits at 5 dpf. **(A)** Contrast response function and **(B)** spatial frequency function from 5 dpf control (closed circles, *n* = 13) and *bbs2^–/–^* mutants (open circles, *n* = 9) plotted as the OKR gain vs. the log contrast. OKR data are from 13 control and 9 mutant larvae. Error bars indicate SEM. Significant differences at individual points were discerned from Sidak’s multiple comparisons tests and indicated with asterisks. Significance levels are as follows: *p < 0.05; ***p < 0.001; ****p < 0.0001.

Visual function was assessed in *bbs2^–/–^* mutant larvae and control siblings (+/+ and + /−) at 5–6 dpf using the optokinetic response (OKR). Larvae were presented with moving stimuli that vary by either contrast or spatial frequency (Rinner et al., 2005). The OKR gain of control (*n* = 13) and *bbs2^–/–^* mutants (*n* = 9) was assessed as previously described (Emran et al., 2010; Daniele et al., 2016; Lessieur et al., 2017). As previously noted (Rinner et al., 2005), the OKR gain is a linear function of the log of contrast in control larvae (Figure 2A; black circles). Relative to control animals, the OKR gain was reduced in *bbs2^–/–^* mutants presented with varying contrast stimuli (Figure 2A; 2-way ANOVA, *p* < 0.0001). When presented with stimuli that varied by spatial frequency, the *bbs2^–/–^* mutants again showed an overall deficit in visual function (Figure 2B; 2-way ANOVA, *p* < 0.005). These results demonstrate that *bbs2^–/–^* larvae exhibit deficits in both general visual function and reduced ability to discriminate spatial differences.

### *bbs2^–/–^* Mutants Have Shorter and Disorganized Photoreceptor Outer Segments

**FIGURE 3:**
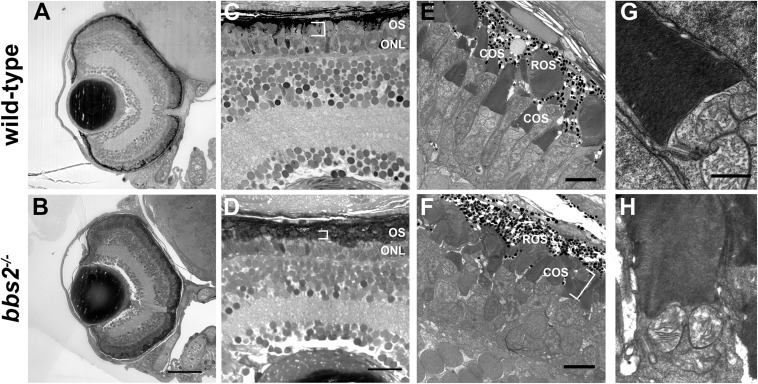
*bbs2 ^– /–^* mutant zebrafish have shortened and disorganized photoreceptor outer segments at 5 dpf. Representative images of wild-type and *bbs2^– /–^* mutants at 5 dpf. **(A,B)** Semi-thin histological sections show that *bbs2^– /–^* mutants have normal retinal lamination. **(C,D)** Higher magnification images of the dorsal retina show that *bbs2^– /–^* mutants have shorter photoreceptor outer segments (OS; white brackets) but gross anatomical structure remains normal. **(E–H)** Transmission electron microscopy shows shorter and disorganized cone outer segments (COS) in *bbs2^– /–^* mutants (white bracket). Cilia architecture appears normal. Scale bar: 50 μm **(A,B)**; 10 μm **(C,D)**; 5 μm **(E,F)**; 1 μm **(G,H)**. COS, outer segments; ROS, rod outer segments.

To determine if the reduced visual function was associated with abnormal retinal anatomy, light microscopy on semi-thin plastic sections and transmission electron microscopy (TEM) was used to assess retinal histology. At 5 dpf, the *bbs2^–/–^* mutants had similar eye size and normal retinal lamination when compared to a wild-type sibling control (Figures 3A,B). Within the photoreceptor layer the outer nuclear layer (ONL) was similar to controls. Within the zebrafish cone mosaic, cones are tiered with red/green double cones residing apically compared to blue-sensitive and UV-sensitive cones (Ramsey and Perkins, 2013). In both semi-thin sections and by transmission electron microscopy (TEM) the cone outer segments (COS) were noticeably shorter in the *bbs2^–/–^* mutants (Figures 3C–F, white brackets). Both wild-type siblings and *bbs2^–/–^* mutants exhibited normal basal body positioning, cilia architecture, and tightly stacked disc membranes within the outer segments (Figures 3G,H). Thus, loss of Bbs2 resulted in shorter photoreceptor outer segments in larval zebrafish.

### Photoreceptors Degenerate in Adult *bbs2^–/–^* Mutants

**FIGURE 4:**
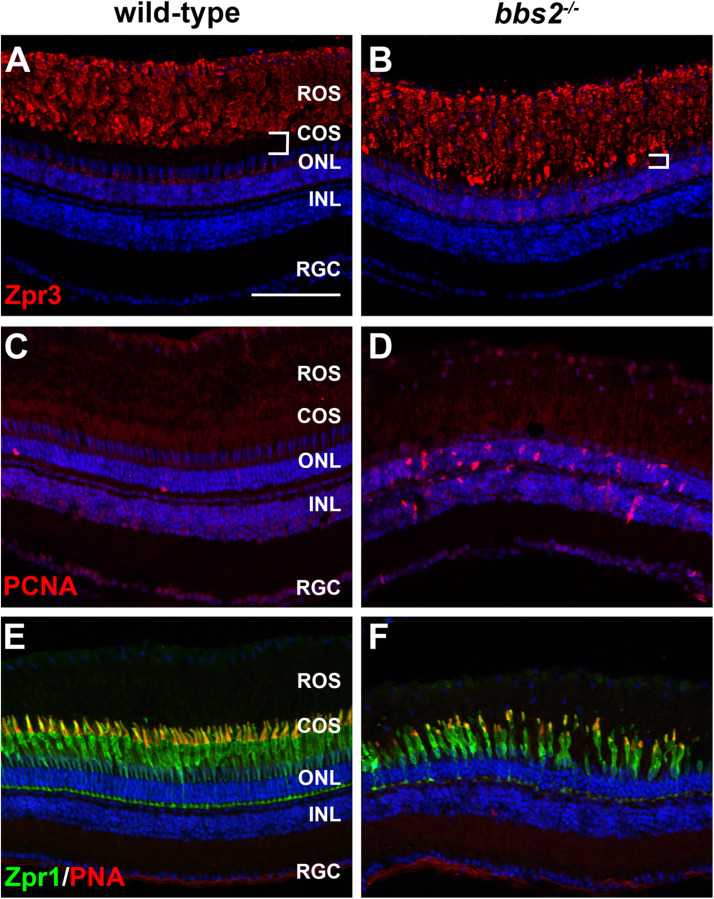
Photoreceptor degeneration in zebrafish retina lacking Bbs2. **(A,B)** Transverse cryosections of 7 mpf wild-type sibling and *bbs2^–/–^* mutant retina stained with Zpr3 (red) to label rhodopsin. The region containing cone outer segments (white brackets) is smaller in *bbs2^–/–^* mutants. **(C,D)** Cryosections of wild-type and *bbs2^–/–^* mutants stained with PCNA (red) to label proliferating cells. **(E,F)** Cryosections of wild-type and *bbs2^–/–^* mutants stained with PNA (red) and Zpr1 (green) to label cone outer segments and cone inner segments, respectively. Sections were counterstained with DAPI (blue). Scale bar: 100 μm. RGC, retinal ganglion cells; INL, inner nuclear layer; ONL, outer nuclear layer; COS, cone outer segment layer; ROS, rod outer segment layer.

We next asked whether the photoreceptor disorganization observed in 5 dpf *bbs2^–/–^* would progress to photoreceptor loss. We previously found obvious rod dysfunction and between 40 and 50% cone loss by 6–7 mpf in *cep290^–/–^* mutants (Lessieur et al., 2019). We therefore examined adult *bbs2^–/–^* mutants at 7 mpf by immunohistochemistry for rod and cone markers and for evidence of Müller glia proliferation. In *bbs2^–/–^* mutants, rhodopsin was mislocalized to the rod inner segments. Although rhodopsin was mislocalized, the size of the rod outer segments did not appear to differ between wild-type and mutant animals (Figures 4A,B). This phenotype resembled that of the zebrafish *cep290^–/–^* mutant, which also showed signs of degeneration but no net loss of rods (Lessieur et al., 2019). Acute retinal injury and cell death triggers the reprogramming and proliferation of Müller glia within the inner nuclear layer (INL) (Goldman, 2014; Wan and Goldman, 2016). In contrast, limited death of rod photoreceptors stimulates proliferation of unipotent rod precursors that reside in the ONL and subsequently differentiate into rod photoreceptors (Stenkamp, 2011). Retinal sections were stained with antibodies against proliferating cell nuclear antigen (PCNA) to identify proliferating cells. In wild-type retinas, only 1–2 cells that stained positive for PCNA were found in the ONL. These likely represent rod precursors, which can also differentiate into new rods as the eye slowly grows in size (Stenkamp, 2011). In contrast, *bbs2^–/–^* zebrafish had numerous PCNA + cells in the ONL and a modest increase in PCNA + cells in the INL (Figures 4C,D). Increased proliferation of unipotent rod precursors in the ONL is consistent with regeneration of dying rod photoreceptors (Morris et al., 2005; Montgomery et al., 2010). Retina cryosections were also co-stained with the zpr-1 antibody, which recognizes arrestin 3a on the red-green double cones (Ile et al., 2010), and with peanut agglutinin (PNA), which labels the extracellular matrix surrounding cone outer segments (Blanks and Johnson, 1984). Compared to wild-type retinas, the *bbs2^–/–^* mutants had fewer cones and the outer segments appeared shorter and more disorganized, suggesting ongoing cone degeneration (Figures 4E,F). The loss of cones also correlated to the reduced distance between the base of the rod outer segments and the ONL (Figures 4A,B, white brackets). Taken together, the data indicate that both rod and cone photoreceptors degenerate; however, rods likely regenerate from the proliferating unipotent rod precursors in the ONL while cones continue to die. The proliferating cells in the INL likely represent those Müller glia that produce rod precursors as a distinct lineage from the multipotent neurogenic progenitors that regenerate cones following acute damage (Stenkamp, 2011).

### Accumulation of Syntaxin-3 in Photoreceptor Outer Segments in Zebrafish Lacking Bbs2

**FIGURE 5:**
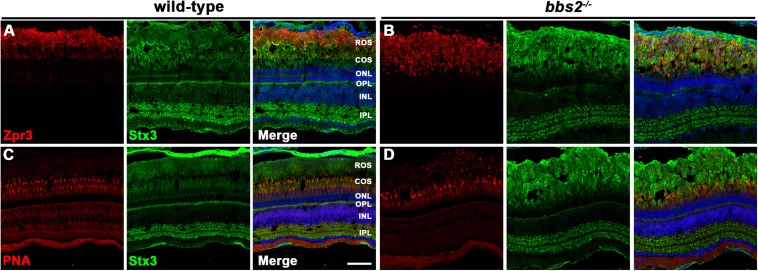
Immunolocalization of syntaxin-3 and cone markers in zebrafish retina lacking Bbs2. **(A,B)** Transverse cryosections of 12 mpf wild-type sibling and *bbs2^– /–^* mutant retina stained with Zpr3 (red) and syntaxin-3 (Stx3; green). **(C,D)** Cryosections of wild-type and *bbs2^– /–^* mutants stained with PNA (red) to label cone outer segments or syntaxin-3 (Stx3; green). Sections were counterstained with DAPI (blue). Scale bar: 50 μm. IPL, inner plexiform layer; INL, inner nuclear layer; OPL, outer plexiform layer; ONL, outer nuclear layer; COS, cone outer segment layer; ROS, rod outer segment layer.

Initially discovered in the *Lztfl1/Bbs17* mouse mutant, a number of studies of *Bbs* mutant mice have showed that the SNARE protein syntaxin-3 (Stx3) accumulates in the photoreceptor outer segments (Datta et al., 2015; Hsu et al., 2017; Dilan et al., 2018) Like many other SNARE proteins, Stx3 normally localizes throughout the inner segments and the synaptic terminals (Kwok et al., 2008). The BBSome is believed to function as a coat adaptor complex between Intraflagellar Transport (IFT) molecules and ciliary cargo, with current evidence suggesting the BBSome ensures exit of ciliary cargo (Liu and Lechtreck, 2018; Nachury, 2018). Thus, the loss of BBSome components prevents proper binding between retrograde IFT trains and ciliary cargo, such as Stx3. The subsequent accumulation of Stx3 in the outer segment may be responsible for photoreceptor degeneration observed in *Bbs* mice (Datta et al., 2015). We chose to examine Stx3 localization in *bbs2^–/–^* mutants at 12 months of age, a time when cone photoreceptor degeneration is significant but rod photoreceptor outer segments remain. The specificity of the polyclonal Stx3 antibodies was validated by *stx3a* morpholino knockdown (Supplementary Figure 1). In wild-type photoreceptors, Stx3 staining was primarily observed in the plexiform layers (Figure 5A and Supplementary Figure 1). In *bbs2^–/–^* mutants, Stx3 accumulated in the rod outer segments and signal from the inner segments and outer plexiform layer was reduced (Figure 5B). Similarly, in wild-type photoreceptors Stx3 did not exhibit strong localization with peanut agglutinin (PNA), a marker for cone outer segments (Figure 5C). Stx3 accumulated in the area of rod outer segments and did not appear enriched in cone outer segments of *bbs2^–/–^* mutants (Figure 5D). These results are consistent with prior studies in mouse and suggest that BBSome function is conserved between zebrafish and mammals.

### Photoreceptor Degeneration Triggers an Inflammatory Response in *bbs2^–/–^* Mutants

**FIGURE 6:**
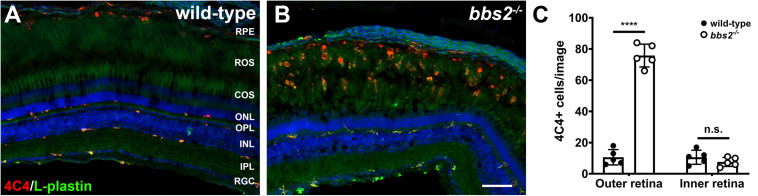
Immunolocalization of immune cells in zebrafish retina lacking Bbs2. **(A,B)** Transverse cryosections of 7 mpf wild-type sibling and *bbs2^– /–^* mutant retina stained with 4C4 (red) and L-plastin (green). Sections were counterstained with DAPI (blue). **(C)** Quantification of 4C4 + microglia per image (1,388 × 1,040 pixels). Each data point represents quantification from individual fish (*n* = 5). ****p < 0.0001. Scale bar: 50 μm. RGC, retinal ganglion cells; IPL, inner plexiform layer; INL, inner nuclear layer; OPL, outer plexiform layer; ONL, outer nuclear layer; COS, cone outer segment layer; ROS, rod outer segment layer; RPE, retinal pigment epithelium.

Acute retinal damage triggers a significant inflammatory response characterized by activated immune cells and reactive Müller glia (White et al., 2017; Mitchell et al., 2018, 2019). To determine if ongoing retinal degeneration in *bbs2^–/–^* mutants was associated with elevated inflammation, retinas from 7 mpf animals were stained with the monoclonal antibody 4C4, which is specific for zebrafish microglia (Tsarouchas et al., 2018; Mazzolini et al., 2020), and L-plastin, a pan-leukocyte marker (Herbomel et al., 1999; Le Guyader et al., 2008). In wild-type adult zebrafish, the 4C4 + microglia maintain a ramified morphology and reside on the basal and apical surfaces of the INL, within the retinal ganglion cells (RGC), and in the outer plexiform layer (Figure 6A). Microglia are also seen in the RPE/choroid. The majority of 4C4 + microglia in the neural retina also stained with L-plastin. Significantly more 4C4 + microglia were observed in the RPE/choroid and the region containing rod and cone outer segments (i.e., outer retina) of 7 mpf *bbs2^–/–^* mutants (Figures 6B,C). Activated microglia appeared amoeboid in shape and largely localized to the outer retina. L-plastin + macrophages also accumulated in the vitreous, suggesting these were infiltrating macrophages responding to degeneration. No morphological changes and no significant numerical increase was observed in the microglia population in the plexiform layers or the INL or GCL (Figure 6C). These data indicate that zebrafish exhibit a robust immune response in response to chronic degeneration. Surprisingly, activated microglia accumulate near outer segments and not in the ONL as is seen in mouse models of retinitis pigmentosa (Zhao et al., 2015).

### Müller Cells of *bbs2^–/–^* Mutants Become Proliferative in Response to Acute Injury

**FIGURE 7:**
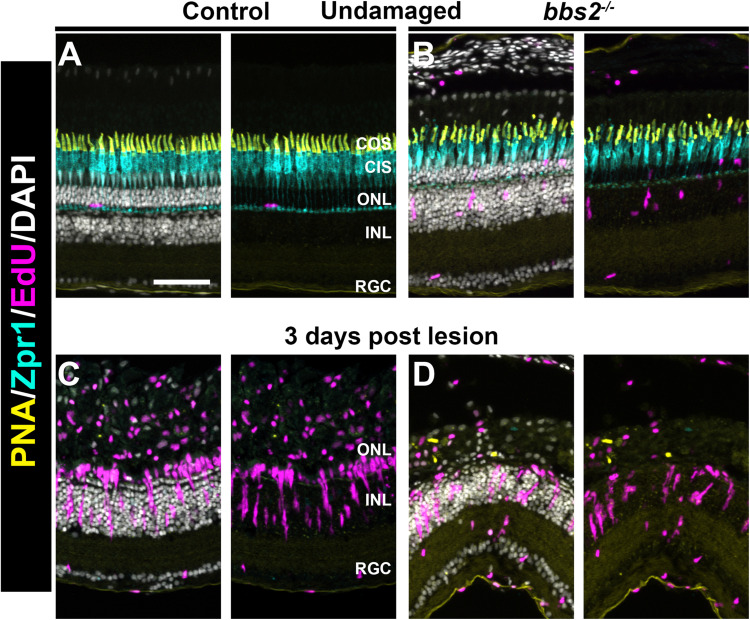
Light damage triggers proliferation of Müller glia in wild-type and *bbs2^– /–^* mutants. Transverse cryosections of undamaged retinas from control **(A)** and *bbs2^– /–^* mutant **(B)** adult (5 mpf). Cryosections were stained with PNA to label cone outer segments (yellow), Zpr1 to stain cone inner segments (cyan), EdU to identify cells that underwent proliferation (magenta) and DAPI (grays). Right panels omit DAPI channel to permit better visualization of EdU labeling. Cone photoreceptors are disorganized and a small number of proliferating cells are seen in the ONL and INL of *bbs2^– /–^* mutants. **(C,D)** Images of transverse cryosections of light damaged control animals **(C)** and *bbs2^– /–^* mutants **(D)** at 3 days post light lesion. Light damage has largely eliminated photoreceptors and neurogenic clusters of EdU + cells are seen in both groups. Scale bar: 50 μm. RGC, retinal ganglion cells; INL, inner nuclear layer; ONL, outer nuclear layer; CIS, Cone inner segments; COS, cone outer segment layer.

In zebrafish, photoreceptor death caused by light damage, mechanical injury, or cytotoxic lesion triggers a robust regeneration response that restores lost photoreceptors (Hyde and Reh, 2014; Wan and Goldman, 2016). As *bbs2^–/–^* mutants undergo progressive degeneration, we wondered whether *bbs2^–/–^* mutants had lost capability to regenerate cones. To address this question, high intensity light was used to cause acute photoreceptor damage (Vihtelic and Hyde, 2000; Thomas and Thummel, 2013). The first steps in regeneration are the de-differentiation and reprogramming of Müller glia and the subsequent proliferation of Müller glia derived progenitor cells. Müller cell proliferation was assessed by EdU incorporation at 3 days post lesion (see section “Materials and Methods”). In undamaged wild-type animals at 5 mpf, little to no proliferation in the ONL or INL was observed (Figure 7A), while some EdU + cells were observed in the ONL and INL of undamaged *bbs2^–/–^* mutants (Figure 7B). At 3 days post lesion (dpl), cone photoreceptors were lost and neurogenic clusters of EdU + cells were observed in the INL of both wild-type and *bbs2^–/–^* mutants (Figures 7C,D). From the position of these proliferating clusters in the INL, it was inferred that these are Müller cell derived retinal progenitors. From these data, we concluded that, like wild-type animals, *bbs2^–/–^* mutants respond to acute injury by stimulating Müller cell proliferation and neurogenesis.

### Photoreceptors Regenerate to Pre-lesion Densities in *bbs2^–/–^* Mutants

**FIGURE 8:**
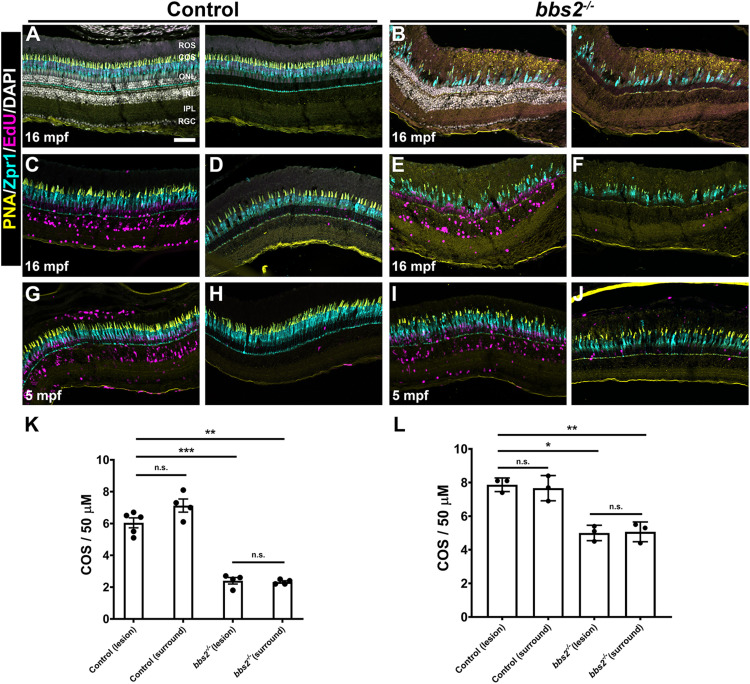
Photoreceptors regenerate to pre-damage density in control and *bbs2^– /–^* mutants. Transverse cryosections of undamaged retinas from control **(A)** and *bbs2^– /–^* mutant **(B)** adults (16 mpf). Cryosections were stained with PNA (yellow), Zpr1 (cyan), EdU (magenta) and DAPI (grays). Right panels omit DAPI channel to permit better visualization of EdU labeling. **(C–J)** Images of transverse cryosections of light damaged 16 mpf control animals **(C,D)** and *bbs2^– /–^* mutants **(E,F)** or 5 mpf control animals **(G,H)** and *bbs2^– /–^* mutants after recovery. Left panels show the regions of the dorsal retina where damage and proliferation occurred (e.g., lesion). Right panels show more distal regions of the dorsal retina that were undamaged (e.g., surround). **(K)** Quantification of cone outer segment (COS) density in lesion and surround areas for 16 mpf control (*n* = 5) and *bbs2^– /–^* mutants (*n* = 4). **(L)** Quantification of cone outer segment (COS) density in lesion and surround areas for 5 mpf control (*n* = 3) and *bbs2^– /–^* mutants (*n* = 3). Each data point represents quantification from an individual animal. *p < 0.01; **p < 0.002. ***p < 0.0001. Scale bar: 50 μm. RGC, retinal ganglion cells; IPL, inner plexiform layer; INL, inner nuclear layer; ONL, outer nuclear layer; COS, cone outer segment layer; ROS, rod outer segment layer.

To determine if neurogenesis in light damaged *bbs2^–/–^* mutants could result in regeneration of cone photoreceptors, animals were allowed to recover for 1 month after light damage. We first tested regeneration in older animals (e.g., 16 mpf) that had lost the majority of cones and where even modest regeneration could be more readily observed. Cone density was quantified in 16 mpf control (wild-type + heterozygous siblings) and *bbs2^–/–^* mutants after 1 month of recovery (Figure 8). In undamaged animals, the number of photoreceptors was significantly reduced in the *bbs2^–/–^* mutants due to ongoing degeneration (Figures 8A,B). Previous groups have demonstrated that exposure to high intensity light results in photoreceptor death and subsequent regeneration primarily in the dorsal retina (Vihtelic et al., 2006; Thomas et al., 2012). Consistent with these observations, we noted that photoreceptor loss 3 days after light damage was limited to the dorsal retina in both control and *bbs2^–/–^* mutants (data not shown). Significant EdU incorporation in the INL and ONL was observed in the dorsal retina at 1 month of recovery (Figures 8C,E). Areas of the same retinas that did not undergo light damage (e.g., “surround”) had few EdU + cells (Figures 8D,F), suggesting limited regeneration. These regions served as internal controls for photoreceptor density and evidence of Müller cell proliferation. To determine if regeneration restored cone density in the lesioned areas of the dorsal retina, the cone outer segment density was quantified in the area containing EdU + cells (e.g., “lesion”) and then compared to the density of cone outer segments in the surround. Following 1 month of recovery, the cones in the lesioned areas of control retinas had regenerated to pre-lesioned levels (Figures 8C,D,K). Cones in the *bbs2^–/–^* retinas also regenerated to pre-lesion densities. However, there were significantly fewer cones both before and after regeneration in *bbs2^–/–^* mutants as compared to control retinas that received light damage (Figures 8E,F,K). Regeneration was also tested in younger adult animals (e.g., 5 mpf) and similar results were observed. EdU + cells were seen throughout the light-damaged region of both control and *bbs2^–/–^* retinas (Figures 8G,I) but not in the undamaged surrounding retina (Figures 8H,J). While the initial density of cones was higher in 5 mpf animals, regeneration restored cones only to pre-lesion levels in the *bbs2^–/–^* mutants (Figure 8L). These data indicate that *bbs2^–/–^* mutants could regenerate damaged cone photoreceptors following acute injury. This ability appears limited, however, to restoring only those cones present at the time damage was incurred, regardless of age. Thus, this partial regeneration was insufficient to restore the *bbs2^–/–^* retina to wild-type cone densities.

## Discussion

The evidence reported here contribute to the broader understanding of BBS pathogenesis across multiple species. As in humans (Shevach et al., 2015) and mice (Nishimura et al., 2004), zebrafish with mutations in *bbs2* undergo a progressive photoreceptor degeneration. Whereas mice lacking other BBSome components, such as Bbs8, show significant degeneration of photoreceptors within a few weeks (Hsu et al., 2017; Dilan et al., 2018), the loss of photoreceptors occurs over months (Nishimura et al., 2004) and humans with *BBS2* mutations may only exhibit mild visual impairment. Our findings that zebrafish *bbs2^–/–^* mutants exhibit progressive degeneration over months is consistent with the rate of degeneration seen in mice and humans. Thus, the affected gene and the nature of the mutations should be considered when evaluating the potential time course of retinal dystrophy in BBS.

Numerous studies of mouse models lacking individual components of the BBSome have established a requirement for the BBSome in photoreceptor morphogenesis and survival (Mykytyn et al., 2004; Abd-El-Barr et al., 2007; Zhang et al., 2014; Datta et al., 2015; Murphy et al., 2015; Dilan et al., 2018). The observed pathology of *bbs2^–/–^* mutants illustrates that BBSome function is conserved in zebrafish. We show that visual function is compromised in larval zebrafish, similar to the ERG defects reported in mice lacking *Bbs4* and *Bbs8* (Abd-El-Barr et al., 2007; Dilan et al., 2018). These visual function defects are associated with abnormalities in rod and cone outer segment morphogenesis in the zebrafish *bbs2^–/–^* mutants. It should be noted, however, that zebrafish vision depends exclusively on cone function during larval stages (Brockerhoff et al., 1995; Bilotta et al., 2001). Thus, the OKR defects in *bbs2^–/–^* zebrafish indicate compromised cone function. This differs from an earlier study that found normal cone function at P30 in mice with a cone-specific knockout of *Bbs8* (Dilan et al., 2018). The zebrafish may, therefore, offer a unique opportunity to investigate the role of Bbs proteins in cone photoreceptor function.

In both mouse and zebrafish, acute retinal injury triggers resident microglia to rapidly migrate and accumulate near the site of injury or disease and release pro-inflammatory cytokines. These microglia release numerous growth factors and inflammatory cytokines that stimulate Müller glia to regenerate lost neurons (Goldman, 2014; Gorsuch and Hyde, 2014; Mitchell et al., 2018, 2019). The cone degeneration observed in zebrafish *bbs2^–/–^* mutants triggers a significant inflammatory response but this is not sufficient to initiate a regenerative response from the Müller glia.

Following light induced retinal damage, we found photoreceptor loss in the dorsal retina of *bbs2^–/–^* mutants, followed by significant Müller glia proliferation in the INL. Taken together, these results suggested that Müller glia do not require Bbs2 function for reprogramming and proliferation and that *bbs2^–/–^* mutants retained the capacity for regeneration following light-induced photoreceptor damage. Although retinal regeneration is not possible in humans, retinal regeneration in zebrafish is to be viewed as a means to develop strategies to treat progressive retinal diseases in humans (Wan and Goldman, 2016). For regeneration to be a viable therapeutic option in advanced cases of retinal disease, restoring photoreceptor density should be a goal. After light damage, the density of regenerated cones within the lesion of *bbs2^–/–^* mutants was similar to that in the neighboring undamaged, but diseased retina. Furthermore, the density of regenerated *bbs2^–/–^* photoreceptors was significantly lower than the density of regenerated wild-type photoreceptors, regardless of the age of the *bbs2^–/–^* mutants. These results suggest that the environment in the degenerated *bbs2^–/–^* retina may not be permissive to a full regenerative response.

These studies contribute to the body of work documenting the essential role of BBS proteins to both rod and cone photoreceptor function across vertebrates. Without normal BBSome function, cone function and photoreceptor outer segment morphogenesis is compromised. The degeneration phenotypes observed in *bbs2^–/–^* zebrafish is similar to that found in zebrafish *cep290^–/–^* and the *eys^–/–^* mutants (Yu et al., 2016; Lessieur et al., 2019). In all three cases, photoreceptors undergo progressive degeneration without significant Müller glia proliferation or regeneration of cones. Our work also illustrates that additional work is needed to more fully understand the relationship between retinal degeneration, inflammation, and the signals required to stimulate Müller glia regeneration.

## Data Availability Statement

The raw data supporting the conclusions of this article will be made available by the authors, without undue reservation.

## Ethics Statement

The animal study was reviewed and approved by Cleveland Clinic Institutional Animal Care and Use Committee (IACUC).

## Author Contributions

PS, JF, and BP conceived and designed experiments. PS, JF, LC, RS, and BP performed experiments and analyzed the data. BP wrote the manuscript. All authors contributed to the article and approved the submitted version.

## Conflict of Interest

The authors declare that the research was conducted in the absence of any commercial or financial relationships that could be construed as a potential conflict of interest.

## Footnotes

1 http://zifit.partners.org/ZiFiT/
