# Supplementary material for: Cone Photoreceptor Degeneration and Neuroinflammation in the Zebrafish Bardet-Biedl Syndrome 2 (bbs2) Mutant Does Not Lead to Retinal Regeneration
Source: Front Cell Dev Biol. 2020 Nov 26;8:578528. doi: 10.3389/fcell.2020.578528 (PMC7726229; doi:10.3389/fcell.2020.578528)
Supplement: Supplementary file 1 [file Data_Sheet_1.docx]

Supplementary Material

**SUPPLEMENTARY MATERIALS AND METHODS**

**Morpholino design and injections**

Morpholinos against *stx3a* were designed and obtained from Gene Tools LLC (<https://www.gene-tools.com/>) and dissolved in water. Splice-blocking morpholinos were designed to target the exon 3 – intron 3 (e3i3) junction (5’ TGTTCGTGTGTCTTACTCTGGTC 3’), the exon 4 – intron 4 (e4i4) junction (5’ ACGTGTAAGGGCACTTACTCTTCAG 3’ ), and the exon 5 – intron 5 (e5i5) junction (5’ TAAGTAGGATGTTGACTCACCTGTG 3’). The standard *tp53* morpholino was also utilized (5’ GCGCCATTGCTTTGCAAGAATTG 3’). Morpholinos were injected into wild-type zebrafish embryos at the 1-cell stage at doses ranging from 0.6 ng – 9 ng per embryo and evaluated every 24 hrs between 1-5 dpf. The translation-blocking morpholino and the e4i4 and e5i5 morpholinos did not result in a phenotype at any dose tested. The e3i3 morpholino resulted in embryos with slightly smaller eyes at 4 dpf. Uninjected embryos and *tp53* morphants were used as controls. Larvae were processed for immunohistochemistry as described in the main text.
