# Supplementary figures and images for: Cone Photoreceptor Degeneration and Neuroinflammation in the Zebrafish Bardet-Biedl Syndrome 2 (bbs2) Mutant Does Not Lead to Retinal Regeneration
Source: Front Cell Dev Biol. 2020 Nov 26;8:578528. doi: 10.3389/fcell.2020.578528 (PMC7726229; doi:10.3389/fcell.2020.578528)

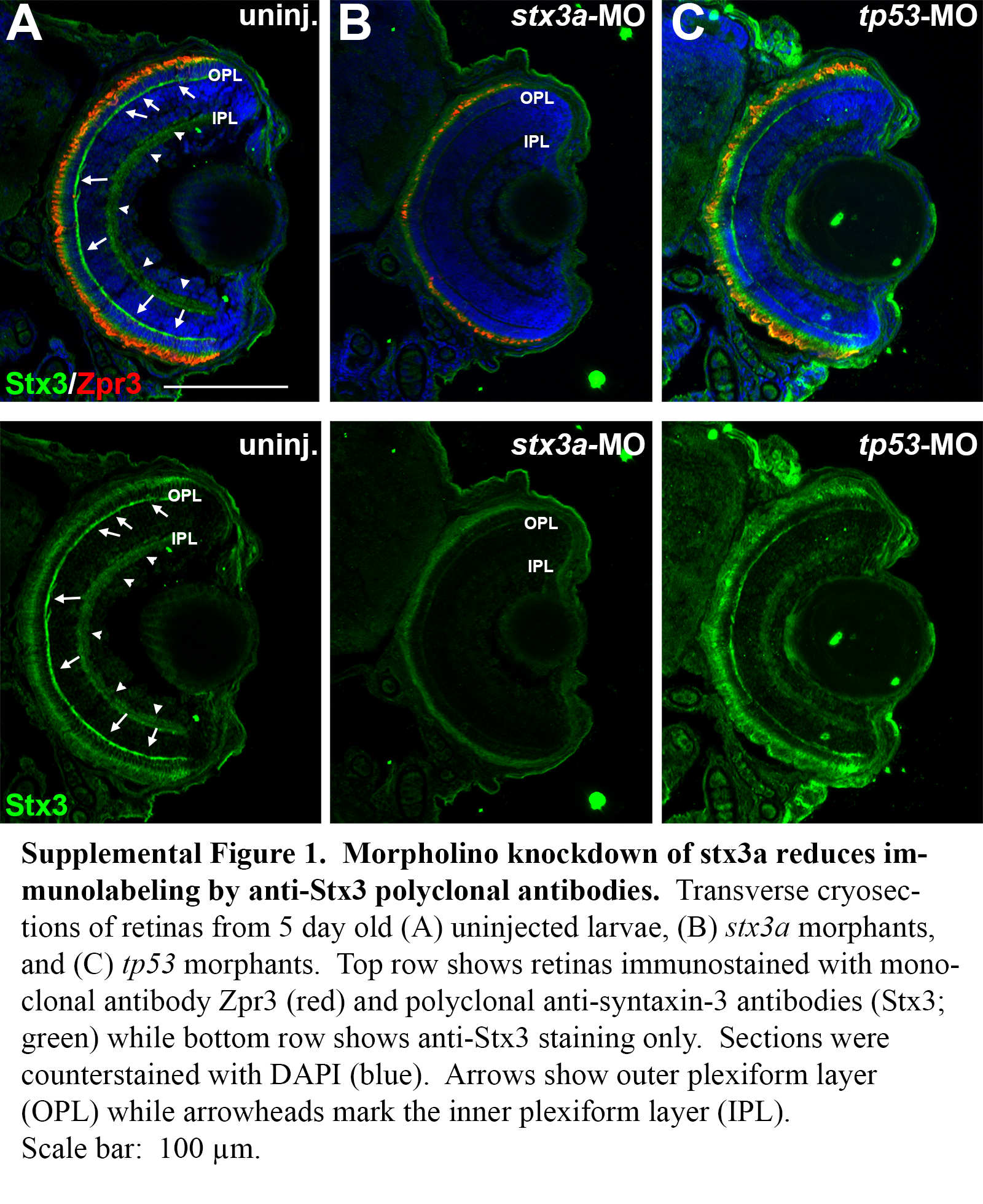

Supplement: Supplementary file 2 [file Image_1.TIF]
